# Induced dicentric chromosome formation promotes genomic rearrangements and tumorigenesis

**DOI:** 10.1007/s10577-013-9368-6

**Published:** 2013-06-22

**Authors:** Karen E. Gascoigne, Iain M. Cheeseman

**Affiliations:** 1 Whitehead Institute for Biomedical Research, Nine Cambridge Center, Cambridge, MA 02142 USA; 2 Department of Biology, Massachusetts Institute of Technology, Cambridge, USA

**Keywords:** Chromosome, Dicentric, Centromere, Kinetochore, Mitosis, Translocation, Genomic rearrangement, Transformation, Cancer

## Abstract

Chromosomal rearrangements can radically alter gene products and their function, driving tumor formation or progression. However, the molecular origins and evolution of such rearrangements are varied and poorly understood, with cancer cells often containing multiple, complex rearrangements. One mechanism that can lead to genomic rearrangements is the formation of a “dicentric” chromosome containing two functional centromeres. Indeed, such dicentric chromosomes have been observed in cancer cells. Here, we tested the ability of a single dicentric chromosome to contribute to genomic instability and neoplastic conversion in vertebrate cells. We developed a system to transiently and reversibly induce dicentric chromosome formation on a single chromosome with high temporal control. We find that induced dicentric chromosomes are frequently damaged and mis-segregated during mitosis, and that this leads to extensive chromosomal rearrangements including translocations with other chromosomes. Populations of pre-neoplastic cells in which a single dicentric chromosome is induced acquire extensive genomic instability and display hallmarks of cellular transformation including anchorage-independent growth in soft agar. Our results suggest that a single dicentric chromosome could contribute to tumor initiation.

## Introduction

Abnormal chromosome and nuclear morphology are key diagnostic characteristics of tumor cells (Hanahan and Weinberg 2000). Almost all cancers show an abnormal and often unstable karyotype, with aneuploid chromosome numbers and the presence of genomic rearrangements including translocations, deletions, fusions, duplications, and insertions. Such rearrangements can radically alter gene products and function, driving tumor formation or progression. For example, the Philadelphia chromosome translocation between chromosomes 9 and 22 produces the Bcr-Abl fusion protein found in most CMLs and is sufficient to drive the disease (Ben-Neriah et al. 1986). Similarly, the MLL transcription factor is commonly found as a fusion protein with multiple partners in AML and is a strong predictor of poor prognosis (Ayton and Cleary 2001). The origins and evolution of such chromosome rearrangements are varied and poorly understood, and cancer cells often contain multiple, complex rearrangements. Previous work has proposed several potential mechanisms for such rearrangements including errors in replication and recombination (Colnaghi et al. 2011), errors in cytokinesis that result in tetraploidy (Fujiwara et al. 2005), errors in centrosome number and function that cause spindle defects (Ganem et al. 2009), telomere erosion (Hemann et al. 2001), and chromosome “shattering” via chromothripsis (Stephens et al. 2011).

An additional mechanism that can lead to genomic rearrangements is the formation of a dicentric chromosome—a chromosome containing two functional centromeres. Indeed, such dicentric chromosomes have been observed in cancer cells, particularly hematological malignancies (Gisselsson et al. 2000; Mackinnon and Campbell 2011; Yamamoto et al. 2011). Such cells often display extensive and complex genome rearrangements. However, the causal link between the presence of a dicentric chromosome and other genome alterations remains unclear. As the centromere provides the site of kinetochore formation and attachment to the microtubule-based mitotic spindle, it is essential that only one functional centromere exist per chromosome. In rare cases, dicentric chromosomes can be stably maintained by inactivating one of the centromeres, but the mechanisms of this inactivation remain poorly understood, occur at low frequency, and are likely to be stochastic (reviewed in Stimpson and Sullivan 2010). However, in many cases, both centromeres remain active and act as building sites for kinetochores during mitosis. The presence of multiple kinetochores on a single chromosome could result in a single sister chromatid simultaneously attaching to both spindle poles, which would cause chromosome mis-segregation or deoxyribonucleic acid (DNA) damage. Spindle-based forces could also generate chromosome breaks such that a dicentric chromosome would be fragmented during mitosis. Chromosome breaks, in turn, often lead to chromosome fusions that alter or dysregulate genes around the break site and could also result in the formation of new dicentric fusion chromosomes. This process of cascading chromosomal damage has been termed the “breakage–fusion–bridge cycle” and is strongly associated with cancer initiation (Cosme-Blanco and Chang 2008; McClintock 1939). Although it has been hypothesized that rearrangements stemming from such cycles are induced by errors in mitosis, this mechanism has not been directly tested at the molecular level in vertebrate cells. In addition, the extent to which a single dicentric chromosome could lead to complex genomic rearrangements and neoplastic transformation has not been explored.

Here, we generated an experimental system to test the effects of assembling two kinetochores on a single chromosome on genome stability and cellular transformation. Our system makes it possible to transiently induce an ectopic kinetochore-like structure at a non-centromeric site on a single chromosome that also contains an endogenous centromere, thereby generating a functionally dicentric chromosome. We demonstrate that induction of a single dicentric chromosome results in chromosome mis-segregation and DNA damage. In subsequent cell divisions, we find that induction of the dicentric chromosome and the corresponding DNA damage results in genomic rearrangements similar to those observed following telomere erosion-induced break–bridge–fusion cycles. The presence of the dicentric chromosome also leads to global genome instability and karyotype abnormalities similar to those found in cancer cells. Finally, induction of the dicentric chromosome in a population of cells leads to the acquisition of characteristics of cellular transformation, suggesting that the presence of a single dicentric chromosome could contribute to tumor initiation.

## Materials and methods

### Cell culture and transfection

Cell lines were maintained as described previously (Kline et al. 2006). NIH3T3 lac operator (lacO) cells (a generous gift from T. Mistelli; Soutoglou et al. 2007) were maintained in 400 μg/ml Hygromycin B. Stable clonal cell lines expressing green fluorescence protein (GFP)-Lac repressor (LacI) fusions were generated as described previously and maintained in 10 mM isopropyl β-d-1-thiogalactopyranoside (IPTG) (Gascoigne et al. 2011).

### Immunofluorescence and microscopy

Immunofluorescence was conducted as described previously (Kline et al. 2006) using anti-phospho-histone H2AX (Millipore) and 53 binding protein 1 (Novus) antibodies. Cy2, Cy3, and Cy5-conjugated secondary antibodies were obtained from Jackson Laboratories. DNA was visualized using 10 μg/ml Hoechst. Images were acquired on a DeltaVision Core deconvolution microscope (Applied Precision) equipped with a CoolSnap HQ2 CCD camera. Z-sections (∼40) for whole cell analysis and ∼10 Z-sections for chromosome spreads were acquired at 0.2 μm steps using a ×100, 1.3 NA Olympus U-Plan Apo objective. Images were deconvolved using the DeltaVision software.

### Fluorescence in situ hybridization and spectral karyotyping

Metaphase chromosome spreads were generated by harvesting cells after 3 h incubation in 100 ng/ml nocodazole, swelling in 75 mM KCl, and fixation in methanol/acetic acid (3:1). Whole chromosome probes to mouse chromosome 3 were obtained from Applied Spectral Imaging (rhodamine labeled FPRPR0169). Probes specific to the lacO array were generated from the pJRC49 LacO plasmid (a kind gift from W Bickmore) and the atto488 nick translation labeling kit (Jena Biosciences) according to the manufacturer’s instructions. Fluorescence in situ hybridization (FISH) probe hybridization was performed overnight at 37 °C as per manufacturer’s instructions. Spectral karyotyping (SKY) analysis was carried out using SKYPAINT^TM^ mouse chromosome probes (Applied Spectral Imaging). Probe hybridization and imaging were carried out by the probe manufacturer.

### Transformation assays

For in vitro soft agar colony-forming assays, a bottom layer of 2 ml 0.5 % agarose in medium was prepared and allowed to solidify. Cells were seeded in a top layer of 0.34 % agarose in medium at a density of 10,000 cells per well in six-well dishes (three wells per treatment). Cells were seeded in the presence of 10 mM IPTG after growth for 4 days in the presence or absence of IPTG as indicated. Cells were incubated at 37 °C and colonies formed after 4 weeks were scored. Invasion assays were carried out in 24-well Transwell® chamber plates containing a Cultrex® basement membrane-coated polycarbonate support with 8 μm pores (Corning). The cells were cultured for 14 days in the presence or absence of 10 mM IPTG as indicated, then seeded into the top chamber at a density of 100,000 cells per chamber. After incubation at 37 °C for the indicated time periods, cells were manually removed from the top chamber and wells were fixed for 10 min in 4 % formaldehyde before staining with 0.1 % crystal violet solution. Cells retained on the bottom side of the membrane were scored at ×10 magnification as cells/field of view.

## Results

### An inducible ectopic kinetochore causes mitotic errors and chromosome damage

To probe the immediate effects of a dicentric chromosome on genome stability and cellular function, we used a previously characterized system (Gascoigne et al. 2011) to induce formation of an ectopic kinetochore-like structure at a non-centromeric site on a single chromosome, thereby generating a functionally dicentric chromosome. Briefly, the N-terminal domain of the key structural kinetochore protein CENP-T was fused to GFP and LacI. Expression of the CENP-T-LacI fusion protein results in the assembly of a kinetochore-like structure at the lacO locus and the interaction of this site with microtubules during mitosis (Gascoigne et al. 2011), whereas expression of the GFP-LacI fusion does not. This ectopic kinetochore structure is capable of rescuing the loss of an endogenous centromere (Gascoigne et al. 2011; Hori et al. 2013), indicating that it is functional to mediate chromosome segregation. Expression of GFP-CENP-T-LacI in cells containing a lacO array leads to assembly of a kinetochore-like structure at the lacO locus. As kinetochore assembly also occurs at the endogenous centromere, this simulates the presence of a dicentric chromosome where two active centromeres nucleate the assembly of two kinetochores on a single chromosome. For the cells used in this study, the position of the lacO array was significantly separated from the endogenous centromere ensuring that both sites of kinetochore assembly function autonomously during mitosis and are capable of interacting independently with microtubules.

**Fig. 1:**
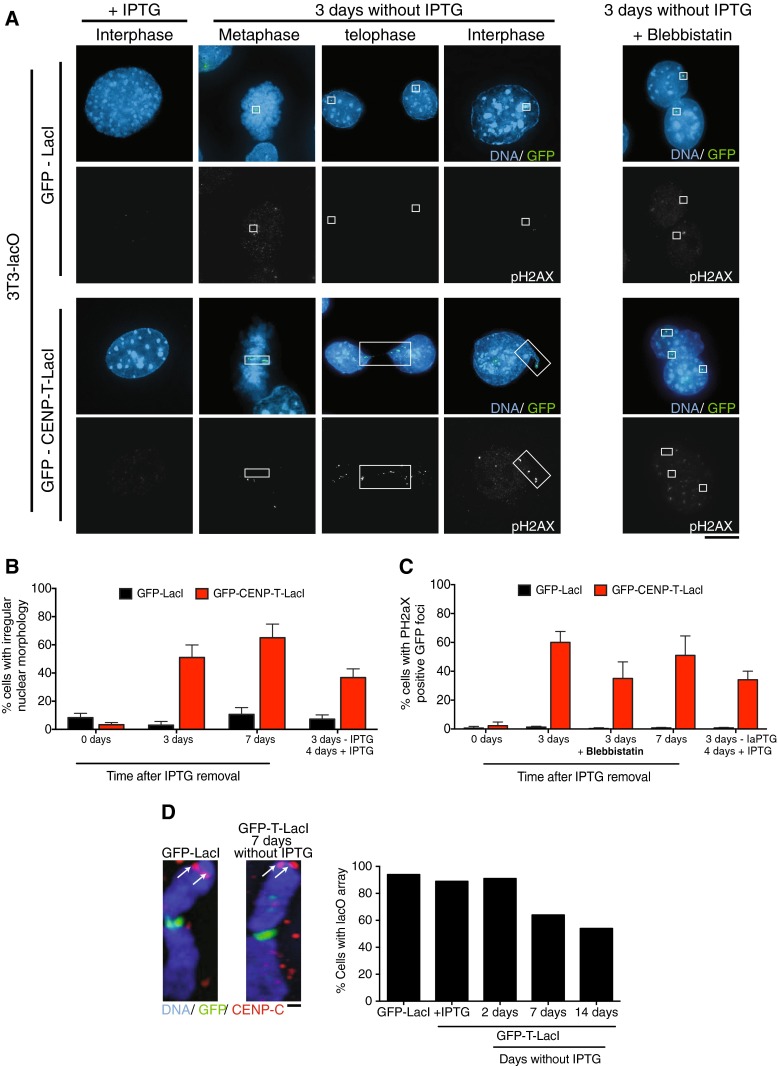
Induction of an ectopic kinetochore causes nuclear abnormalities and DNA damage in 3T3-LacO cells. **a** Stable cell lines expressing GFP-CENP-T ΔC-LacI or GFP-LacI were generated in 3T3 cells containing a lacO array on chromosome 3. Growth in the presence of 10 mM IPTG prevented LacI–lacO interactions. Representative immunofluorescence images show 3T3-LacO cells after removal of IPTG for 3 days and staining with anti-phospho-H2AX antibodies. Distortion of the GFP-CENP-T-LacI containing chromosome is clearly seen after removal of IPTG, as well as co-localization of DNA damage signal with GFP-CENP-T-LacI. *Right panel* shows nuclear morphology after removal of IPTG and growth in 30 μM blebbistatin, indicating reduction in nuclear abnormalities after inhibition of cytokinesis. *Boxes* indicate the location of the GFP signal. *Scale bar* shows 5 μm. **b** Quantification of the number of 3T3-lacO cells with irregular nuclear morphology (including nuclear protrusions, multi-lobed nuclei, and separated nuclei connected by chromatin bridges) in the presence of the indicated LacI fusion protein and following removal of IPTG for 3 or 7 days, or removal for 3 days, then re-addition for 4 days. **c** Quantification of the number of 3T3-lacO cells with phospo-H2AX signals co-localized with GFP foci in immunofluorescence images after growth in the indicated conditions. For **b** and **c**; *n* ≥ 100 cells in three independent experiments. *Error bars* show standard deviation. **d** Shows representative images of LacO (*green*) containing chromosomes that also have an active endogenous centromere in the indicated conditions, as detected by CENP-C immunofluorescence staining in *red*. *Arrows* indicate CENP-C-specific staining. *Scale bar* shows 1 μm. *Graph* shows the number of 3T3-LacO cells containing the lacO array in the indicated conditions

**Fig. 2:**
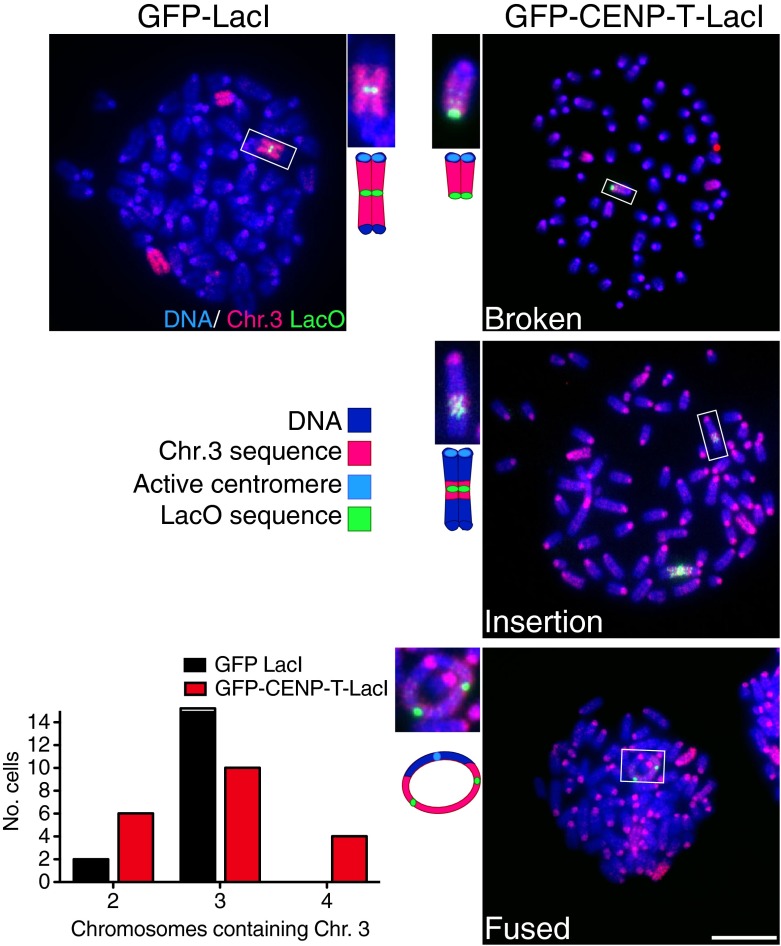
Induction of an ectopic kinetochore causes genomic rearrangements. 3T3-lacO cells expressing GFP-LacI or GFP-CENP-T-ΔC-LacI were grown in the absence of IPTG for 3 days and then prepared for FISH analysis using probes against chromosome 3 (*pink*) and LacO sequence (*green*). Images show representative metaphase spreads after FISH. Note that nonspecific background signal from chromosome 3 probes is also visible on centromeres. *Scale bar* shows 10 μm. *Insets* show examples of chromosome rearrangements. Graph shows quantification of the number of chromosomes containing chromosome 3 sequences. *N* > 15 cells per treatment

For this study, we stably expressed the GFP-CENP-T-LacI fusion in mouse NIH3T3 cells in which an array of lacO repeats was integrated into chromosome 3 (Soutoglou et al. 2007) (Fig. 1a). 3T3 cells are a non-transformed immortalized fibroblast cell line. The 3T3-lacO parental cell line used in this study displayed contact inhibition and anchorage-dependent growth consistent with the lack of transformation. Although we observed moderate whole chromosome aneuploidy in these cells, we did not observe spontaneous genomic rearrangements, indicating the 3T3 cells are stable at the intra-chromosomal level as has been characterized previously (Leibiger et al. 2013). The 3T3-lacO cell line contains a modal number of three copies of chromosome 3 per cell (Fig. 2). One of these copies consists of two copies of chromosome 3 fused together at their q arms by an array of lac operator repeats, forming a structurally dicentric, but functionally monocentric chromosome (Soutoglou et al. 2007). One centromere on this chromosome is inactive, producing a single structure with an active centromere at one end of the chromosome and an array of lacO repeats at the center (Fig. 2).

To prevent prolonged ectopic kinetochore formation during normal cell growth, we maintained the 3T3-lacO cells stably expressing GFP-CENP-T-LacI in the presence of 10 mM IPTG to disrupt the lacO/LacI interaction. In this way, ectopic kinetochore and dicentric chromosome formation could be induced with high temporal control by removal of IPTG from the culture media (Fig. 1a). After removal of IPTG, GFP focus formation at the lacO locus was observed after 8 h. However, we noted some variability in the time required for GFP foci to be visible in mitotic cells after IPTG removal (data not shown). Therefore, to ensure that the majority of cells in the observed population had undergone a mitotic division in the presence of the ectopic kinetochores, we observed cells at least 3 days after removal of IPTG (Fig. 1a).

The presence of multiple kinetochore structures on a single chromosome could result in a single sister chromatid simultaneously attaching to both spindle poles causing chromosome mis-segregation or DNA damage. To monitor the immediate effects of dicentric chromosome induction, we performed an immunofluorescence analysis of cells following the removal of IPTG for 3 days. As described previously, following IPTG removal and the induction of the ectopic kinetochore-like structure, the CENP-T-LacI focus became distorted in mitosis suggesting the presence of microtubule interactions (Fig. 1a; Gascoigne et al. 2011). As previously described for U2OS cells containing CENP-T-LacI/lacO foci (Gascoigne et al. 2011), in 3T3 cells, we observed mis-segregation of the lacO-containing chromosome during anaphase and a high proportion of cells with chromosome bridges at telophase (Fig. 1a). Following mitosis in the presence of the ectopic kinetochore, we observed a high frequency of cells with multiple fragmented GFP foci and irregular nuclear morphology. For these cells, instead of the smooth circular morphology of the 3T3 cell nucleus, we observed shape alterations including multi-lobed nuclei, nuclear protrusions, and separated nuclei connected by chromosome bridges (Fig. 1a, b; in Fig. 1a, lower panel telophase shows an example of a chromosome bridge, and the interphase cell shows a nuclear protrusion). In contrast, control cells expressing GFP-LacI showed normal telophase morphology and a single GFP focus per cell (Fig. 1b). In vivo, mammalian cells often stabilize a dicentric chromosome by inactivating one of the endogenous centromeres (Earnshaw and Migeon 1985; Higgins et al. 1999; Page et al. 1995; Sullivan and Schwartz 1995). To test the consequences of transient exposure to a dicentric chromosome in our system, we observed cells grown for 3 days in the absence of IPTG to induce the dicentric chromosome, followed by growth for 4 days in the presence of IPTG to inactivate the ectopic kinetochore. Using these conditions, we observed a slight reduction in the number of nuclear abnormalities observed compared to cells analyzed directly after 3 days of growth in the presence of the dicentric chromosome (Fig. 1b). This suggests that some recovery in nuclear organization can occur following inactivation of the dicentric chromosome.

To assess whether the abnormalities we observed in the GFP-CENP-T-LacI expressing cells resulted in DNA damage, we stained cells with antibodies recognizing the DNA damage markers phospho-histone-H2AX (p-H2AX; Fig. 1a, c) and 53 binding protein 1 (53BP1). In all cases, the anti-p-HA2X signal co-localized with the anti-53BP1 signal (data not shown). After division in the presence of an induced ectopic kinetochore, p-H2AX staining was observed at GFP-CENP-T-LacI foci (Fig. 1a, c). In contrast, control cells expressing GFP-LacI did not show detectable DNA damage at the GFP focus. The level of DNA damage was slightly reduced 4 days after re-addition of IPTG to inactivate the induced dicentric chromosome (Fig. 1c), suggesting that repair of this damage and recovery of the cell are possible once the dicentric chromosome is inactivated. Interestingly, DNA damage following cell division in the presence of the dicentric chromosome could be reduced by the addition of the myosin inhibitor blebbistatin to prevent cytokinesis, suggesting that some of the observed damage may be due to cytokinetic forces acting on DNA trapped in the cleavage furrow during telophase (Fig. 1a).

Although the CENP-T-LacI expressing 3T3-lacO cells showed chromosome mis-segregation and DNA damage, we did not observe significant cell death or senescence within the population following induction of the kinetochore-like structure. One possibility to explain this is that the dicentric chromosome was altered in some way to allow it to be stably maintained. For example, deletion of one centromere on a dicentric chromosome has been observed in response to dicentric chromosome formation in yeast, allowing survival in a small fraction of the population (Bloom et al. 1989; Jager and Philippsen 1989; Sato et al. 2012). Survival occurs more frequently in mammalian cells, where inactivation of one centromere on a dicentric chromosome is commonly observed (Earnshaw and Migeon 1985; Higgins et al. 1999; Page et al. 1995; Sullivan and Schwartz 1995). However, in our system, the endogenous centromere on the dicentric chromosome remained active, even after prolonged removal of IPTG, as indicated by the presence of normal levels of the kinetochore protein CENP-C (Fig. 1d), suggesting that centromere inactivation is not responsible for survival of these cells. Instead, we observed loss of the lacO-containing chromosome over time (Fig. 1d), which would be tolerated in this cell line due to the presence of two additional copies of chromosome 3.

Taken together, these observations suggest that the induction of a dicentric chromosome leads to DNA damage and abnormalities in nuclear structure.

### Inducible dicentric chromosomes undergo chromosome translocations and lead to genomic instability

To assess the specific nuclear changes caused by the induction of a single dicentric chromosome in vertebrate cells, we carried out a karyotype analysis of cells following transient induction of the dicentric chromosome. We first performed FISH analysis to identify chromosome 3 (containing the LacO array) in mitotic spreads from 3T3-lacO cells expressing GFP-LacI or GFP-CENP-T-LacI. We note that nonspecific fluorescence signals from the chromosome 3 probes are also visible at the centromeres of all chromosomes, which can be distinguished by their size, position at the end of the chromosome, and co-localization with the heterochromatin at the centromere, which stains brightly with DAPI. Control 3T3-lacO cells contained a modal number of three chromosomes staining positive for chromosome 3 sequences per cell (Fig. 2). One of these chromosomes was noticeably larger than the other two and stained positive for lacO sequence, indicating it is the lacO-containing chromosome (Fig. 2). Following induction of the ectopic kinetochore on chromosome 3, we observed an increase in the number of cells with more than three chromosomes containing chromosome 3 sequences. In many cases, these additional sequences were not whole chromosome additions, but instead represented insertion of chromosome 3 sequences into another chromosome (Fig. 2). Although control 3T3 cells and GFP-LacI expressing cells did not display chromosome rearrangements involving chromosome 3, following IPTG washout, induced dicentric cells displayed a wide variety of rearrangements involving chromosome 3 both in *cis* including fusions of two dicentric chromosomes, as well as in *trans* such that sections of the dicentric chromosome became translocated with other chromosomes. We note that dicentric ring fusions observed could also be the result of incomplete replication.

**Fig. 3:**
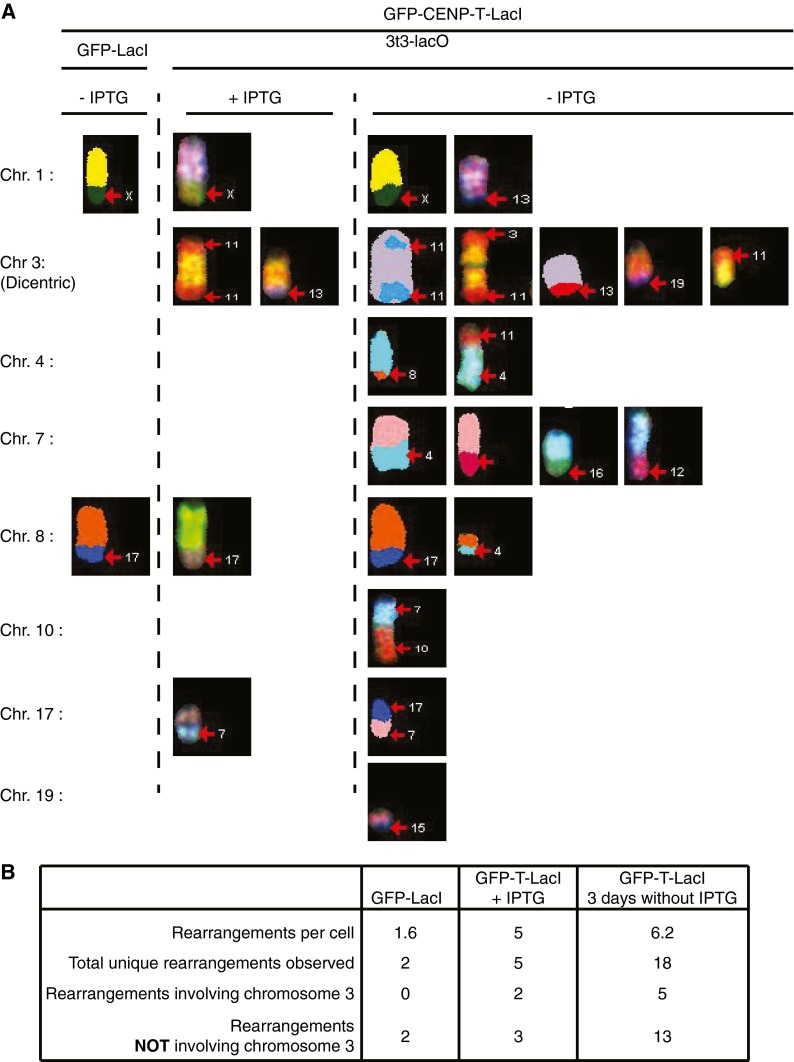
Dicentric chromosome induction leads to global genomic rearrangement. **a** Spectral karyotype (SKY) analysis of 3T3-lacO cells expressing GFP-LacI or GFP-CENP-T-ΔC-LacI at a time point 3 days after removal of IPTG. Representative images of chromosomes involved in translocations are pseudo-colored. **b** Quantification of the rates of chromosome translocations in **a**

In addition to these results using chromosome 3-specific probes, we conducted SKY analysis to visualize the entire range of chromosome sequences in 3T3-lacO cells following dicentric chromosome induction (Fig. 3). In addition to translocations involving the induced dicentric chromosome (chromosome 3), this analysis revealed an increased frequency of rearrangement events not involving chromosome 3 following induction of the dicentric chromosome (Fig. 3a, b). The 3T3-lacO cell line used in this study contained two stable rearrangements: t(1:X) and t(8:17) (Soutoglou et al. 2007). Clonal 3T3-lacO cell lines expressing CENP-T-LacI generated in this background, but grown in the presence of IPTG, contained three additional stable rearrangements (Fig. 3a) likely formed during selection of this clone. However, these rearrangements appeared to be very stable as they were detected in virtually all cells analyzed, without additional rearrangement occurring. Importantly, following induction of the dicentric chromosome, 13 additional rearrangements were observed, ten of which did not involve chromosome 3 (Fig. 3b). Distinct patterns of translocations were observed in individual cells, and on average, each induced dicentric cell analyzed contained at least one additional unique translocation. This pattern of translocations likely reflects the occurrence of random events rather than selection of stable clones in the population. These data suggest the presence of an underlying genomic instability following dicentric chromosome induction. Taken together, these data suggest that the dicentric chromosome can be damaged and broken during mitosis, and that the broken chromosome ends are repaired by fusion and translocation with other chromosomes. In addition, such events appear to cause global genome instability, leading to a variety of genomic rearrangements involving different chromosomes.

## Transient dicentric chromosome induction leads to characteristics of cellular transformation

**Fig. 4:**
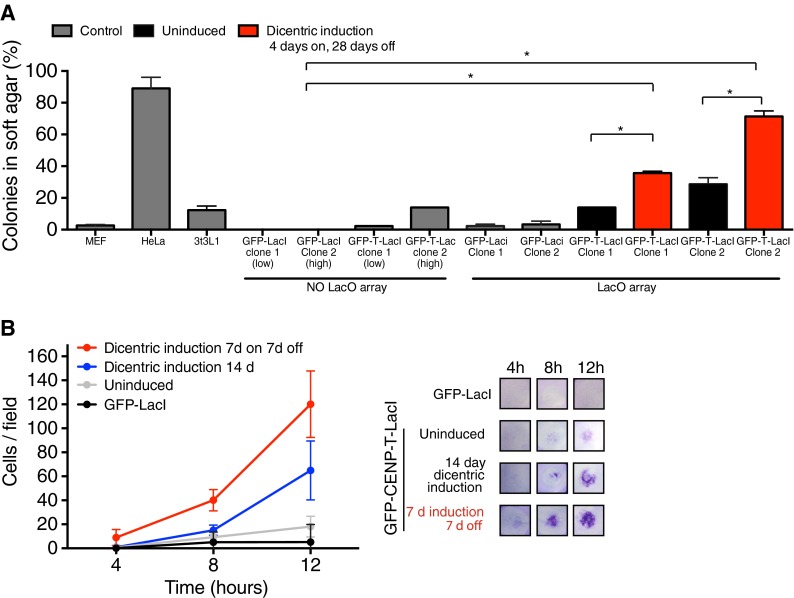
Induction of an ectopic kinetochore causes cellular transformation. **a** 3T3 cells with or without an integrated lacO array and expressing GFP-LacI or GFP-CENP-T-ΔC-LacI were grown for 4 days in the presence or absence of IPTG as indicated. Cells were then plated in triplicate in soft agarose in the presence of IPTG. After 4 weeks of growth, 200 cells were counted per replicate, and the percentage of cells forming colonies was scored. Clonal cell lines expressing (high) or (low) levels of the fusion protein were tested for each condition as indicated. Primary mouse embryonic fibroblasts (*MEFs*) and HeLa cells were used as negative and positive controls, respectively. *Asterisk* indicates significant difference as determined by *T* test, *P* < 0.001. **b** 3T3-lacO cells expressing GFP-LacI or GFP-CENP-T-ΔC-LacI were grown for 14 days in the presence or absence of IPTG as indicated. Cells were then seeded into the upper chamber of a basement membrane-coated Boyden’s chamber and migration across the membrane was assayed at the indicated time points by staining with crystal violet. *N* = 20 fields. *Error bars* show standard deviation. *Right hand panel* shows images of lower membrane staining from a representative experiment

To test the hypothesis that the genomic rearrangements caused by a dicentric chromosome could contribute to cellular transformation, we analyzed 3T3-lacO cells for markers of transformation following dicentric chromosome induction. Growth in soft agar is an established assay to measure the extent of neoplastic transformation in cultured cells (Freedman and Shin 1974). 3T3 cells showed limited colony-forming ability in soft agar (Fig. 4a). However, following transient induction of the dicentric chromosome by growth for 4 days in the absence of IPTG followed by re-addition of IPTG for 28 days, 3T3-lacO cells expressing GFP-CENP-T-LacI displayed a significantly increased capability for anchorage-independent growth compared to control cells expressing GFP-LacI (Fig. 4a). Interestingly, the growth soft agar was reduced for 3T3-lacO/GFP-CENP-T-LacI cells grown continually in the absence of IPTG to induce the dicentric chromosome for the duration of the experiments (32 days; data not shown). This suggests that transient exposure to the dicentric chromosome maybe particularly transformative, but that the long-term presence of the dicentric chromosome may disrupt cell growth and behavior. Although expression of GFP-CENP-T-LacI only slightly increased the colony-forming ability in the absence of the lacO array, GFP-CENP-T-LacI/lacO cells grown continually in the presence of IPTG displayed enhanced growth in soft agar compared to controls, suggesting that basal LacI/lacO interactions may occur resulting in dicentric chromosome formation at low frequency (Fig. 4a).

We also assessed the invasive potential of the induced dicentric 3T3 cells by assessing migration across an extracellular matrix barrier, a common characteristic of cellular transformation (Albini et al. 1987). Cells grown in the presence of the dicentric chromosome were significantly more invasive than control cells in this assay (Fig. 4b). After 8 h of growth, significant migration across the barrier was observed in 3T3-lacO cells expressing the CENP-T-LacI fusion, relative to GFP-LacI control cells. Again, transient induction of the dicentric chromosome led to greater invasive capacity compared to continual exposure for the duration of the experiment (Fig. 4b). Taken together, these data strongly suggest that induction of a single dicentric chromosome can lead to cellular transformation and the acquisition of tumorigenic potential.

## Discussion

Here, we describe an experimental system to model dicentric chromosome formation with high temporal control, allowing us to dissect the consequences of this physiologically relevant chromosome abnormality on genome stability and cellular function. By rapid induction of a functional ectopic kinetochore on the arm of a single chromosome, we monitored the immediate effects of two attachment sites to the mitotic spindle. We found that in non-transformed 3T3-lacO cells, the induced dicentric chromosome was often mis-segregated and became damaged after mitosis (Fig. 1). This damage could be reduced by inhibition of cytokinesis, suggesting cytokinetic forces may be partly responsible for damage to chromosomes trapped in the cleave furrow during telophase, as has been described previously (Janssen et al. 2011). When we analyzed the karyotype of cells grown in the presence of the dicentric chromosome, we observed a variety of chromosome rearrangements involving the dicentric chromosome, both in *cis* and in *trans* (Figs. 2 and 3). Finally, after transient growth in the presence of the dicentric chromosome, populations of 3T3-LacO cells showed a significantly enhanced capacity for invasion and anchorage-independent growth, indicating the acquisition of a transformed phenotype (Fig. 4). In vivo, it is likely that cells rapidly stabilize a dicentric chromosome by inactivating one of the endogenous centromeres and so are only transiently exposed to the consequences of an active dicentric (Earnshaw and Migeon 1985; Higgins et al. 1999; Page et al. 1995; Sullivan and Schwartz 1995). However, when we mimicked this subsequent inactivation in our system by transient induction of the ectopic kinetochore followed by re-addition of IPTG, cells showed greatly enhanced transformation relative to those continually exposed to the dicentric chromosome (Fig. 4). This suggests that a pulse of genome instability caused by an active dicentric may be sufficient to induce transformation.

The data presented here are consistent with the classic “breakage–fusion–bridge” model proposed over 70 years ago (McClintock 1939), but which until recently had been untested at a molecular level in vertebrate cells. Dicentric chromosomes have been observed in a number of physiological situations and can arise by several different mechanisms. For example, telomere erosion can lead to chromosome fusions producing dicentrics and subsequently result in tumor formation (Blasco et al. 1997; Davoli and de Lange 2012). Similarly, translocation events that occur spontaneously or by other mechanisms such as errors in replication or recombination could also result in dicentric chromosomes. However, it was unclear if the presence of a single dicentric chromosome could drive global genome instability and tumorigenesis, or if dicentric chromosomes are instead a consequence of other alterations that occur within the cell during the transformation process. Here, we showed that a single dicentric chromosome undergoing mis-segregation during mitosis can lead to extensive genomic rearrangements similar to those found in cancer cells. The observation that a dicentric chromosome can induce rearrangements in other monocentric chromosomes suggests that the presence of the dicentric may induce an underlying genomic instability, for example, due to abnormalities in the completion of mitosis. Consistent with this, errors in chromosome segregation have recently been shown to drive structural rearrangements in addition to whole chromosome aneuploidy (Crasta et al. 2012; Janssen et al. 2011). Our data are also consistent with analysis of telomere dysfunction in yeast, where global genome instability is rapidly induced as a result of loss of telomere function or after recombination-mediated generation of a dicentric chromosome (Hackett et al. 2001; Hackett and Greider 2003). Taken together, these data describe the molecular fate of a dicentric chromosome during and following the completion of mitosis and indicate an important role for such chromosome aberrations in the initiation of genomic instability and cancer. In the future, it will be important to analyze the types and patterns of genomic aberrations caused by a dicentric chromosome and how they contribute to altered cellular functions.

## Abbreviations

CML: Chronic myeloid leukemia
MLL: Mixed lineage leukemia
AML: Acute myeloid leukemia
DNA: Deoxyribonucleic acid
GFP: Green fluorescence protein
LacI: Lac repressor
lacO: Lac operator
IPTG: Isopropyl β-d-1-thiogalactopyranoside
p-H2AX: Phospho-histone-H2AX
53BP1: 53 binding protein 1
FISH: Fluorescence in situ hybridization
SKY: Spectral karyotype
